# Supplementary material for: Dataflow programming for the analysis of molecular dynamics with AViS, an analysis and visualization software application
Source: PLoS One. 2020 Apr 21;15(4):e0231714. doi: 10.1371/journal.pone.0231714 (PMC7173788; doi:10.1371/journal.pone.0231714)
Supplement: S1 Table — Custom nodes can be implemented by the user to interact with internal nodes, regardless of language. (PDF) [file pone.0231714.s001.pdf]

S1 Table. Examples of in-built nodes provided by AViS. Custom nodes can be implemented by the user to interact with internal nodes, regardless of language.

Particle Data	coordinates and trajectories of all atoms
System Info	configuration information of the loaded system
Get / Set Attribute	Read / Write per-atom attribute data
Set Radius Scale	Scale the radius of atoms
Show Range	Show / Hide atoms based on unique values
Add Bonds	Draw additional atom-atom bonds
Draw Surface	Draw an equi-value 3D surface
To Adjacency / Paired List	Converts the data storage format for connectivity lists
Plot Data	Plots a graph of arbitrary data
