# Supplementary material for: Dataflow programming for the analysis of molecular dynamics with AViS, an analysis and visualization software application
Source: PLoS One. 2020 Apr 21;15(4):e0231714. doi: 10.1371/journal.pone.0231714 (PMC7173788; doi:10.1371/journal.pone.0231714)
Supplement: S1 Appendix — (PDF) [file pone.0231714.s002.pdf]

S1 Appendix. a valid fortran analysis script that generates a sine wave

```
module makesin
    use iso_c_binding
    implicit none
!@in
    integer :: count = 0
!@in
    real*8 :: scale = 0
!@out
    real*8, allocatable, target :: result(:)
    contains
!@entry
    subroutine execute()
        integer :: a
        if (allocated(result)) deallocate(result)
        allocate(result(1:count))
        do a = 1, count
            result(a) = sin(a*scale)
        end do
    end subroutine execute
end module makesin
```
