# Supplementary material for: Dataflow programming for the analysis of molecular dynamics with AViS, an analysis and visualization software application
Source: PLoS One. 2020 Apr 21;15(4):e0231714. doi: 10.1371/journal.pone.0231714 (PMC7173788; doi:10.1371/journal.pone.0231714)
Supplement: S2 Appendix — (PDF) [file pone.0231714.s003.pdf]

S2 Appendix. A valid Python analysis script that differentiates an array

```
import numpy as np

#@in list(1d)
arr = np.zeros(1)
#@out list(1d)
res = np.zeros(1)

#@entry
def Do():
    global arr, res
    res = np.diff(arr)
```
