# Supplementary material for: Dataflow programming for the analysis of molecular dynamics with AViS, an analysis and visualization software application
Source: PLoS One. 2020 Apr 21;15(4):e0231714. doi: 10.1371/journal.pone.0231714 (PMC7173788; doi:10.1371/journal.pone.0231714)
Supplement: S3 Appendix — (PDF) [file pone.0231714.s004.pdf]

S3 Appendix. A valid C++ analysis script that applies an exponential decay to a signal

```cpp
#include <cmath>

//@in cnt
double* array = 0;
//@in
double mag = 0;
//@out cnt
double* out = 0;
//@var
int cnt = 0;

//@entry
void execute() {
    if (out) delete[](out);
    out = new double[cnt];
    for (int a = 0; a < cnt; a++) {
        out[a] = array[a] * std::exp(-a * mag);
    }
}
```
