# Supplementary material for: Dataflow programming for the analysis of molecular dynamics with AViS, an analysis and visualization software application
Source: PLoS One. 2020 Apr 21;15(4):e0231714. doi: 10.1371/journal.pone.0231714 (PMC7173788; doi:10.1371/journal.pone.0231714)
Supplement: S4 Appendix — (PDF) [file pone.0231714.s005.pdf]

S4 Appendix. Comment syntax for Fortran scripts

```
!all comments start with '!@'
!input variables are declared with 'in'
!output variables are declared with 'out'
!supported types are integer*2, integer, real*8

!a scalar variable
!@in
integer*2 :: foo = 0
!@in
integer :: bar = 0

!an array variable is an allocatable
!@out
real*8, allocatable, target :: baz (:,:)

!the function to be executed is declared with 'entry'
!@entry
subroutine exec()
```
