# Supplementary material for: Dataflow programming for the analysis of molecular dynamics with AViS, an analysis and visualization software application
Source: PLoS One. 2020 Apr 21;15(4):e0231714. doi: 10.1371/journal.pone.0231714 (PMC7173788; doi:10.1371/journal.pone.0231714)
Supplement: S5 Appendix — (PDF) [file pone.0231714.s006.pdf]

S5 Appendix. Comment syntax for Python scripts

```
#arrays are declared with numpy
import numpy as np

#all comments start with '#@'
#input variables are declared with 'in'
#output variables are declared with 'out'
#the type of variable must be specified after the declaration

#a scalar variable
#supported types: short, int, double
#@in int
foo = 0
#@out double
bar = 1.0

#an array variable is a numpy ndarray
#type is 'list(nt)' where n=dimension and t=first character of the type (s, i, d)
#@out list(1d)
baz = numpy.ones(5)
#@out list(2s)
qux = numpy.zeros((1, 1), dtype=np.int16)

#the function to be executed is declared with 'entry'
#@entry
def Exec():
```
