# Supplementary material for: Dataflow programming for the analysis of molecular dynamics with AViS, an analysis and visualization software application
Source: PLoS One. 2020 Apr 21;15(4):e0231714. doi: 10.1371/journal.pone.0231714 (PMC7173788; doi:10.1371/journal.pone.0231714)
Supplement: S6 Appendix — (PDF) [file pone.0231714.s007.pdf]

S6 Appendix. Comment syntax for C++ scripts

```
//all comments start with '//@'
//input variables are declared with 'in'
//output variables are declared with 'out'

//a scalar variable
//supported types are short, int, double
//@in
int foo = 0;
//@out
double bar = 1.0;

//an array variable is a pointer
//for each dimension, an int variable holding the size must be specified
//a 2-dimensional array, the element at (a, b) is [ny * a + b]
//@out nx, ny
double* baz = new double[4];

//for each variable used as the array size holder,
//if the variable is not used as input or output,
//it must be declared with 'var'
//@out
int nx = 2;
//@var
int ny = 2;

//the function to be executed is declared with 'entry'
//@entry
void Exec() {
```
