# Supplementary material for: Dataflow programming for the analysis of molecular dynamics with AViS, an analysis and visualization software application
Source: PLoS One. 2020 Apr 21;15(4):e0231714. doi: 10.1371/journal.pone.0231714 (PMC7173788; doi:10.1371/journal.pone.0231714)
Supplement: S7 Appendix — Each entry in the first line represents the type of data in each column. (PDF) [file pone.0231714.s008.pdf]

S7 Appendix. An example of a SSV file with rotational and potential attributes. Each entry in the first line represents the type of data in each column.

```
# posx posy posz attr=rotx attr=roty attr=rotz attr=potential
1000
    0.0  1.0  0.5  0.0        1.0        0.0        −0.1
    0.2  0.8  0.3  1.0        0.0        0.0        −0.2
...
```
