# Supplementary material for: Dataflow programming for the analysis of molecular dynamics with AViS, an analysis and visualization software application
Source: PLoS One. 2020 Apr 21;15(4):e0231714. doi: 10.1371/journal.pone.0231714 (PMC7173788; doi:10.1371/journal.pone.0231714)
Supplement: S1 Fig — AViS handles data transfer between Fortran, Python, and C++ scripts automatically, including arrays as seen here. (PDF) [file pone.0231714.s009.pdf]

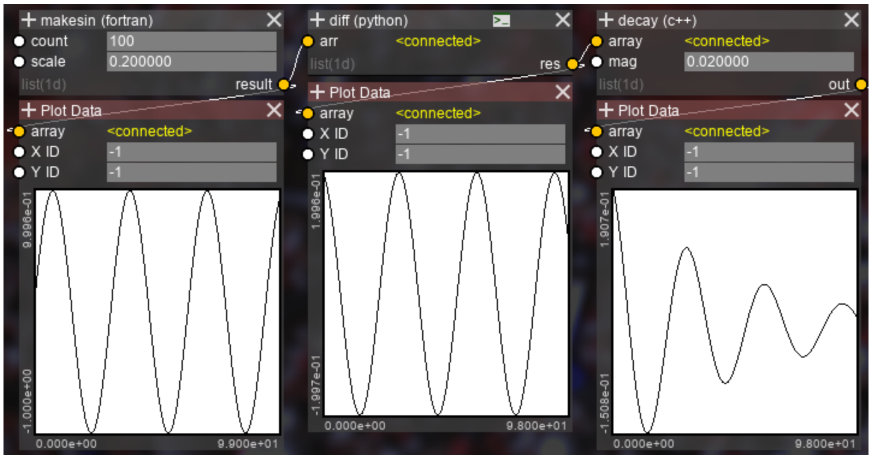

S1 Fig. Multi-language support for analysis. AViS handles data transfer between Fortran, Python, and C++ scripts automatically, including arrays as seen here.
