# Supplementary material for: Dataflow programming for the analysis of molecular dynamics with AViS, an analysis and visualization software application
Source: PLoS One. 2020 Apr 21;15(4):e0231714. doi: 10.1371/journal.pone.0231714 (PMC7173788; doi:10.1371/journal.pone.0231714)
Supplement: S2 Fig — The molecular data of the loaded configuration and trajectory can be accessed using the input nodes. The positions and velocities of the current visible frame, as well as of the whole trajectory can be accessed, as well as additional attributes that are imported, if available. The input values update to the data for each frame over the trajectory when the graph is executed for all frames. (PDF) [file pone.0231714.s010.pdf]

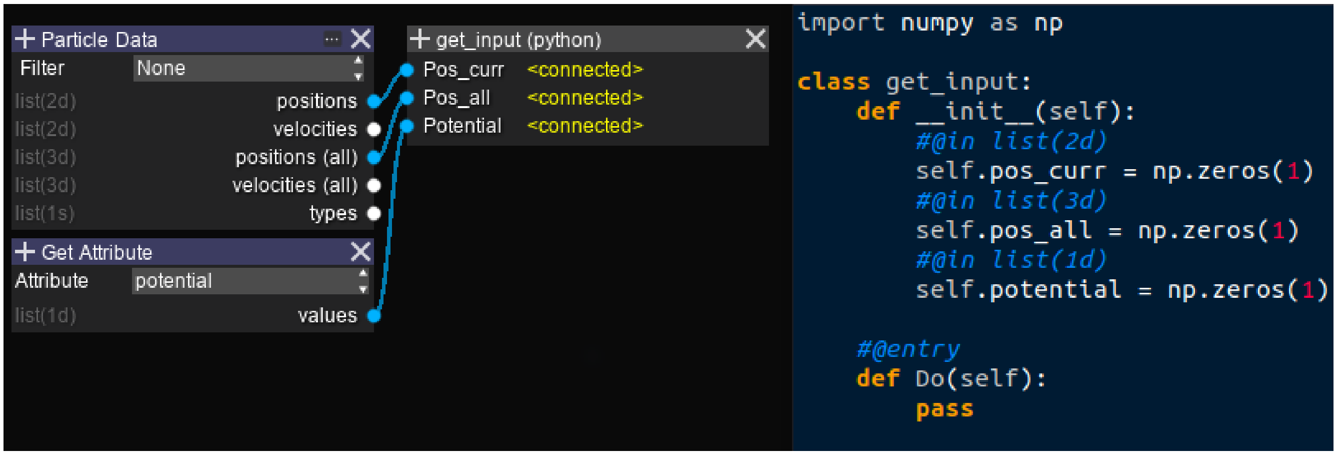

S2 Fig. Accessing molecular data from scripts. The molecular data of the loaded configuration and trajectory can be accessed using the input nodes. The positions and velocities of the current visible frame, as well as of the whole trajectory can be accessed, as well as additional attributes that are imported, if available. The input values update to the data for each frame over the trajectory when the graph is executed for all frames.
