# Supplementary material for: Dataflow programming for the analysis of molecular dynamics with AViS, an analysis and visualization software application
Source: PLoS One. 2020 Apr 21;15(4):e0231714. doi: 10.1371/journal.pone.0231714 (PMC7173788; doi:10.1371/journal.pone.0231714)
Supplement: S3 Fig — (a) By importing the orientation data as attributes with the Generic SSV format, liquid crystal molecules can be visualized without the need to write a custom plugin or importer. In this figure, a color gradient is also applied based on the roty attribute. (b) Individual attributes can be used in analysis by utilizing the Get Attribute node. (PDF) [file pone.0231714.s011.pdf]

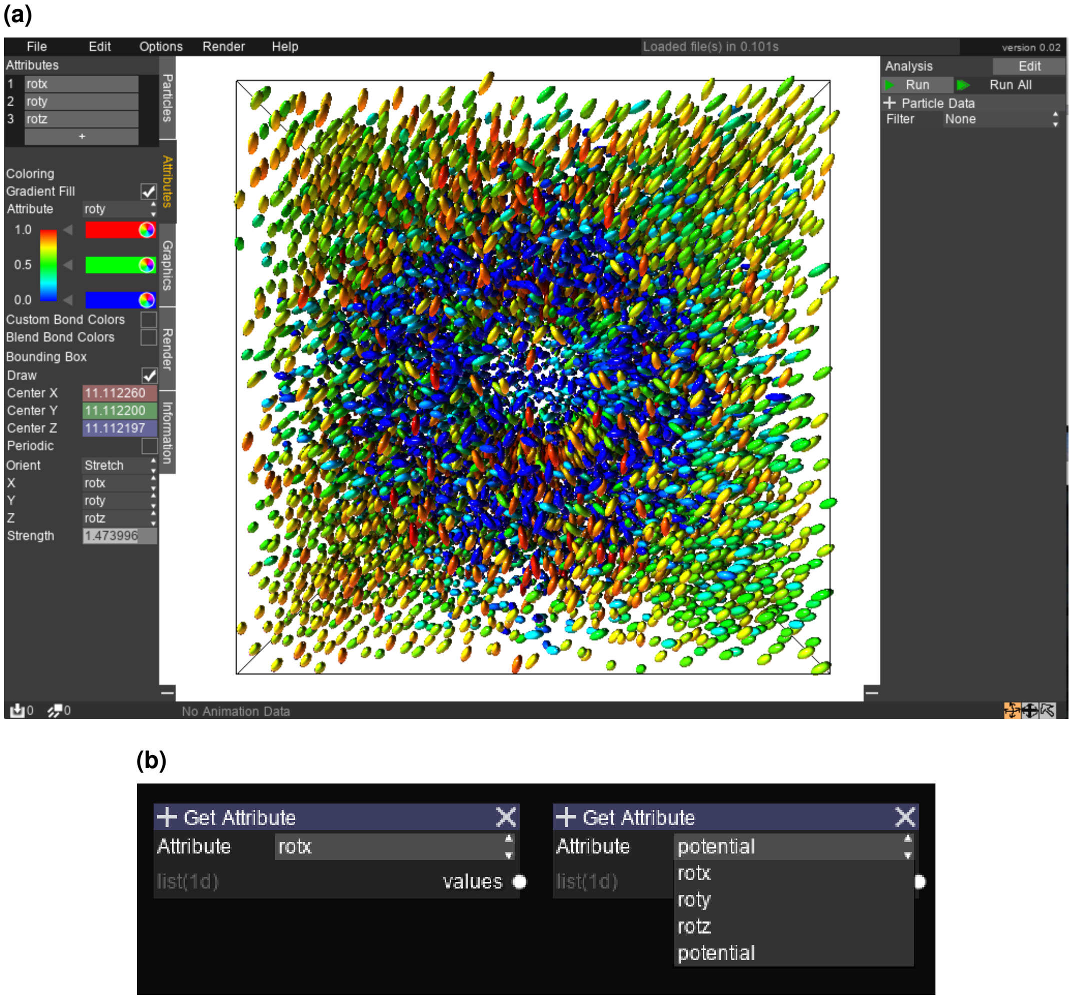

S3 Fig. Visualization and analysis of molecules with attribute data. (a) By importing the orientation data as attributes with the Generic SSV format, liquid crystal molecules can be visualized without the need to write a custom plugin or importer. In this figure, a color gradient is also applied based on the *roty* attribute. (b) Individual attributes can be used in analysis by utilizing the *Get Attribute* node.
