# Supplementary material for: Dataflow programming for the analysis of molecular dynamics with AViS, an analysis and visualization software application
Source: PLoS One. 2020 Apr 21;15(4):e0231714. doi: 10.1371/journal.pone.0231714 (PMC7173788; doi:10.1371/journal.pone.0231714)
Supplement: S4 Fig — (a) Bounding quads are generated using a vertex shader. (b)(c) Surface information channels are generated using ray-tracing in a fragment shader. (d) A Physically-based Rendering (PBR) shader combines the channels into the final image. (e) By utilizing deferred shading, UI overlays and image effects can be added without re-drawing the whole scene, thus improving performance. (PDF) [file pone.0231714.s012.pdf]

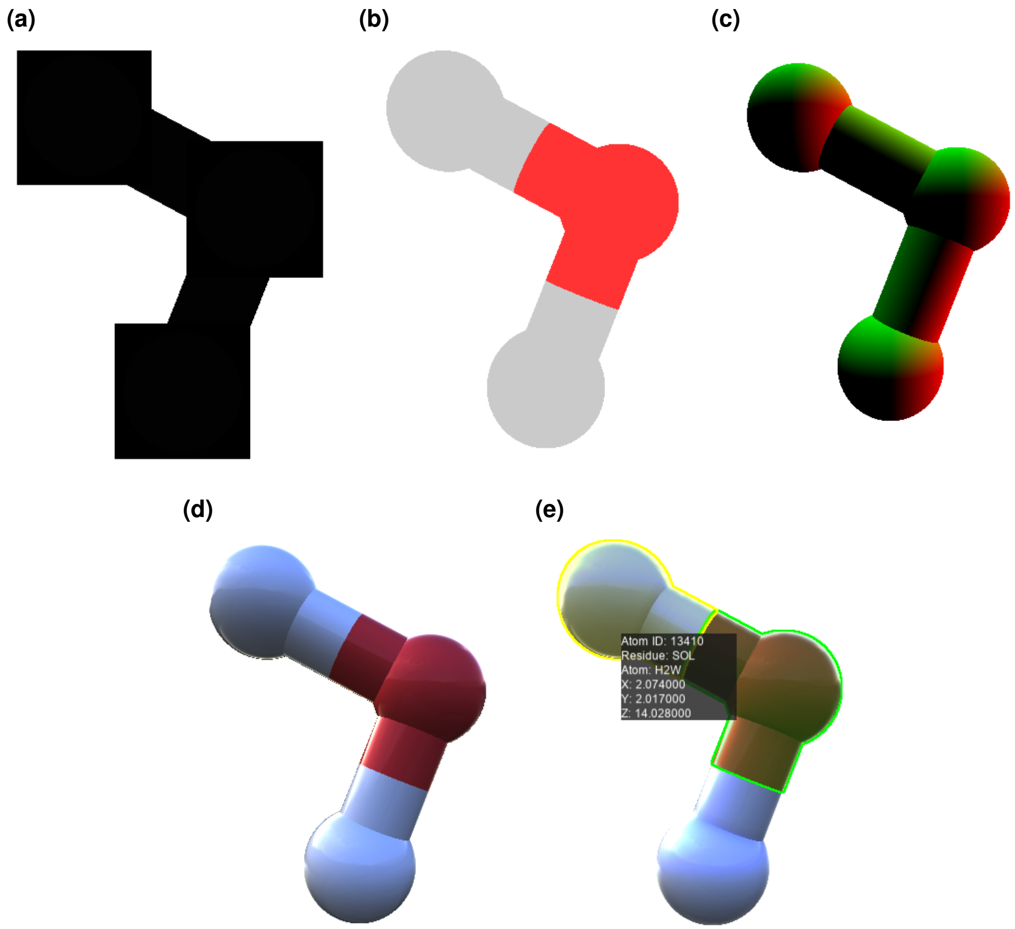

S4 Fig. The rendering procedure. (a) Bounding quads are generated using a vertex shader. (b)(c) Surface information channels are generated using ray-tracing in a fragment shader. (d) A Physically-based Rendering (PBR) shader combines the channels into the final image. (e) By utilizing deferred shading, UI overlays and image effects can be added without re-drawing the whole scene, thus improving performance.
