# Supplementary material for: Dataflow programming for the analysis of molecular dynamics with AViS, an analysis and visualization software application
Source: PLoS One. 2020 Apr 21;15(4):e0231714. doi: 10.1371/journal.pone.0231714 (PMC7173788; doi:10.1371/journal.pone.0231714)
Supplement: S5 Fig — (a) The current frame. (b) The next frame. (c) The number of particles belonging to the previous cluster is counted. (d) The labels are updated for the cluster with the largest count. (PDF) [file pone.0231714.s013.pdf]

(a) (b) (c) (d)

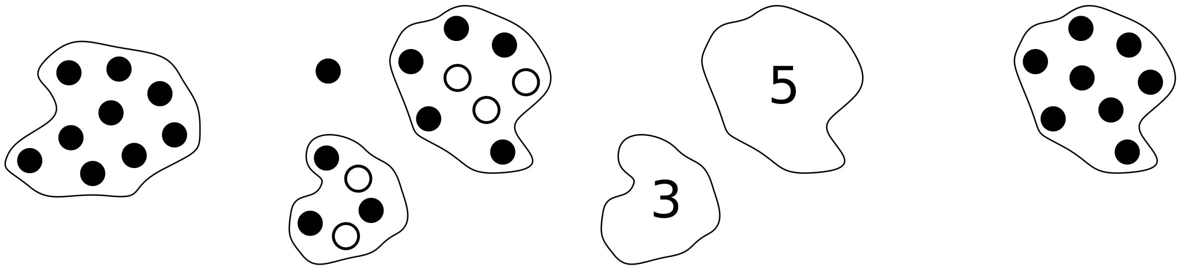

S5 Fig. The visual representation of the tracking algorithm. (a) The current frame. (b) The next frame. (c) The number of particles belonging to the previous cluster is counted. (d) The labels are updated for the cluster with the largest count.
