# Supplementary material for: Dataflow programming for the analysis of molecular dynamics with AViS, an analysis and visualization software application
Source: PLoS One. 2020 Apr 21;15(4):e0231714. doi: 10.1371/journal.pone.0231714 (PMC7173788; doi:10.1371/journal.pone.0231714)
Supplement: S6 Fig — A visual representation of the mutually coordinated guest order parameter used in the case study on clathrate hydrates. The algorithm is presented by Barnes et al. [17]). (PDF) [file pone.0231714.s014.pdf]

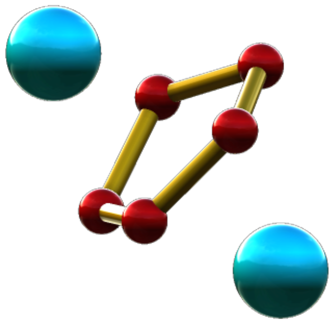

S6 Fig. Representation of the mutually coordinated guest order parameter used in the case study on clathrate hydrates. The algorithm is presented by Barnes *et al.*([16] in main text).
