# Supplementary material for: Dataflow programming for the analysis of molecular dynamics with AViS, an analysis and visualization software application
Source: PLoS One. 2020 Apr 21;15(4):e0231714. doi: 10.1371/journal.pone.0231714 (PMC7173788; doi:10.1371/journal.pone.0231714)
Supplement: S7 Fig — By recording the number of classified molecules every step, we can identify the point of hydrate formation at about frame 2000. (PDF) [file pone.0231714.s015.pdf]

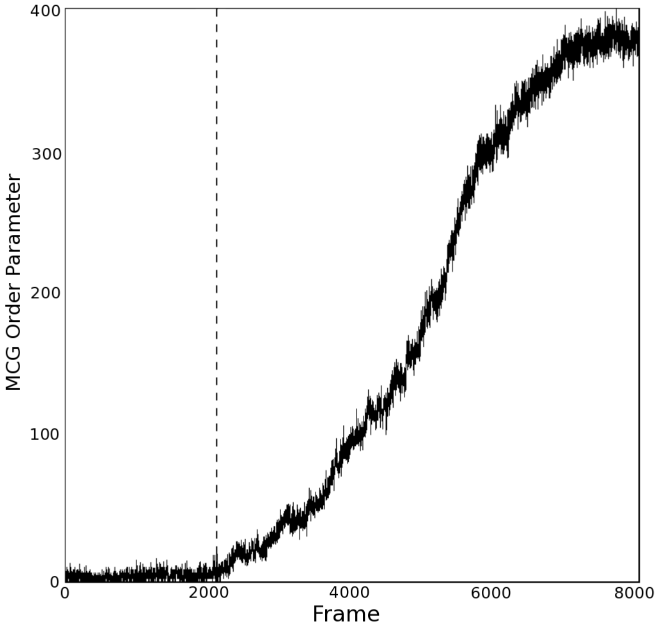

S7 Fig. The value of the order parameter across the whole trajectory. From this graph, we can identify the point of hydrate formation at about frame 2000.
