# Supplementary material for: Dataflow programming for the analysis of molecular dynamics with AViS, an analysis and visualization software application
Source: PLoS One. 2020 Apr 21;15(4):e0231714. doi: 10.1371/journal.pone.0231714 (PMC7173788; doi:10.1371/journal.pone.0231714)
Supplement: S1 File — (ZIP) [file pone.0231714.s016.zip › scr/howtorecreate.pdf]

How to recreate the case studies

This document shows how to recreate the analysis shown in the paper, using the scripts and trajectories provided in this archive. For this example, the LJ Nucleation case study is used. The Hydrate Nucleation case study can also be done with the same steps.

1 Data preparation

Extract the script files from `nucleation/cpp/` to the ~`/.avis/nodes/nucleation/` folder. Optionally replace .cpp files with .f90 or .py from the respective folders.

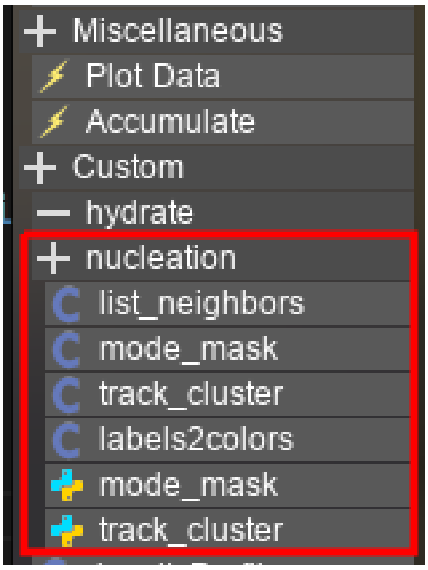

Open the example trajectory with `File/Import`:

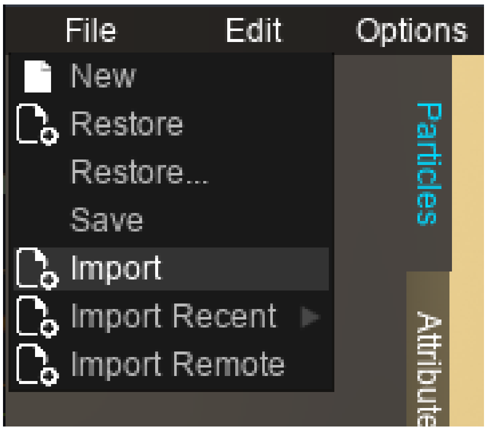

As the file in split into frames, open only the first file. The latter files will be loaded automatically.

2 Graph recreation

Given this graph (Fig. 2a) from the text:

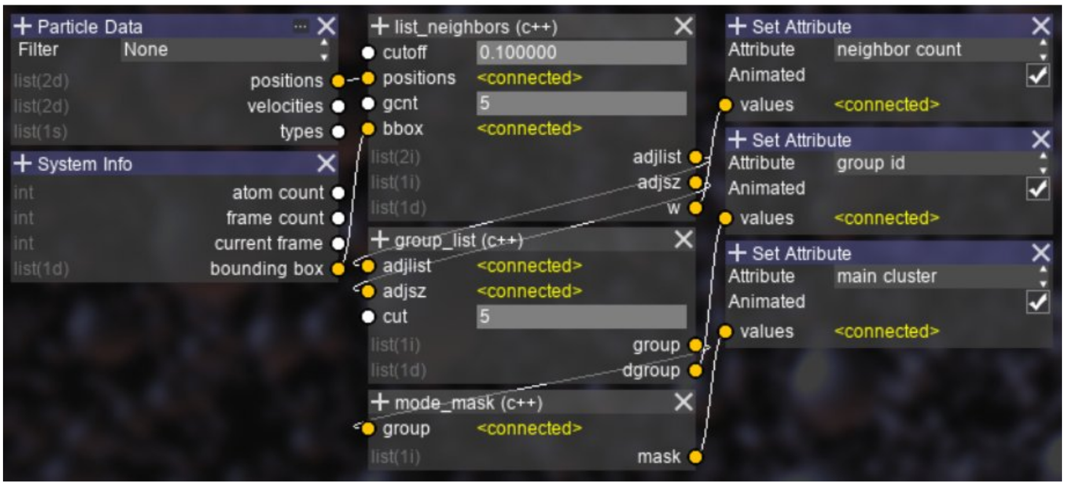

Open the analysis window from the top right:

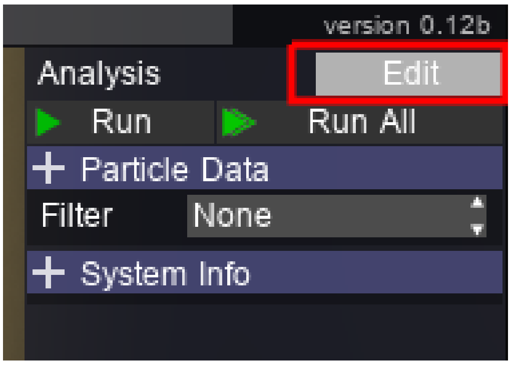

Return to the visualization window similarly by the top right button.

Place the `list_neighbors`, `group_list`, and `mode_mask` scripts, and the `Set Attribute` script from the Writers tab onto the center space.

Click on a script:

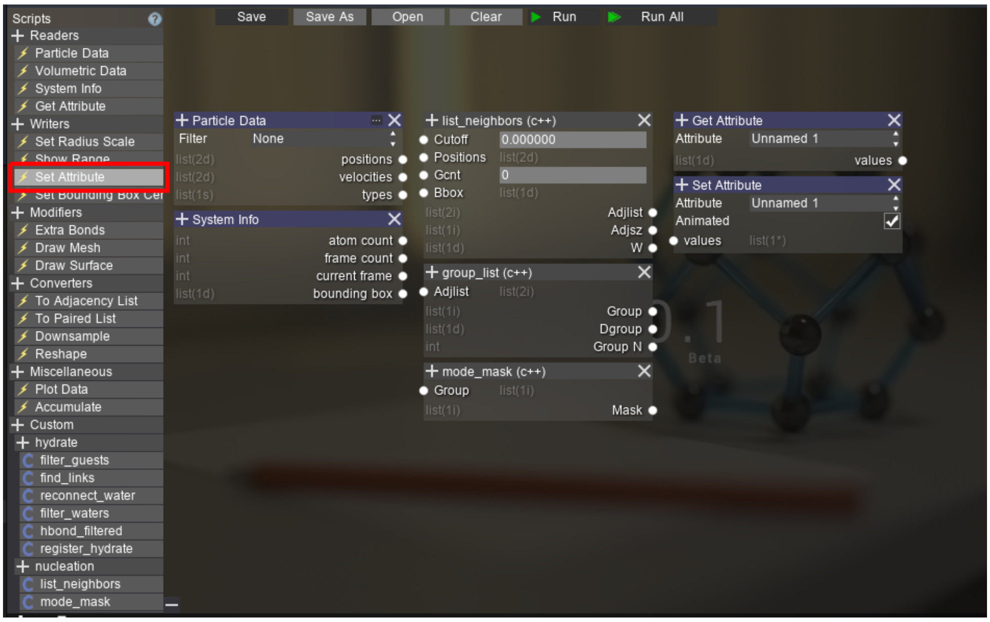

and click on an available space ("+" region):

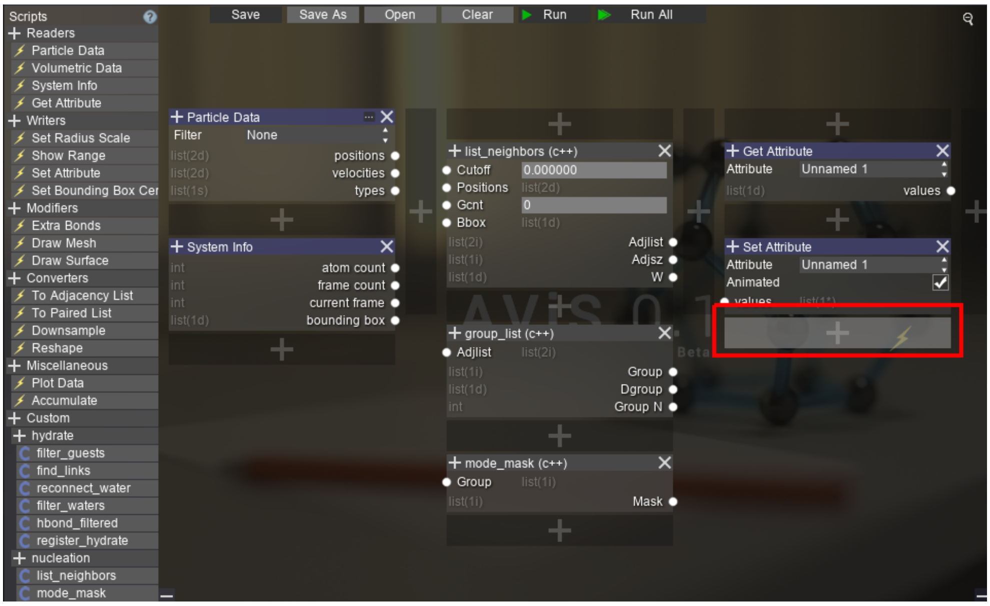

Next, connect the data lines to match the figure. For example, as the `list_neighbors` node requires the particle positions, connect the positions output of `Particle Data` to the positions input. Click on an output's circle:

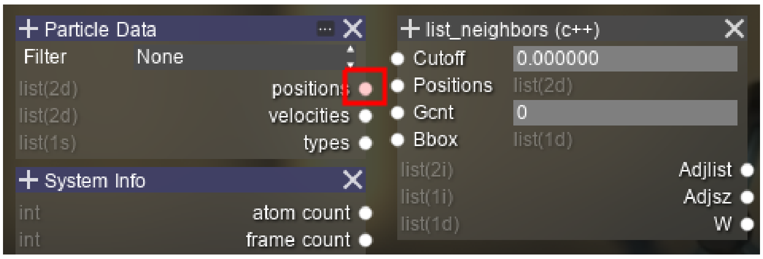

and click on an input's circle:

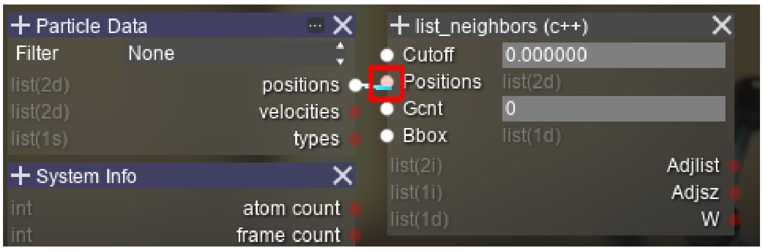

Connect all the required lines similarly. For non-connected inputs, manually enter the values onto the fields, such as `0.1` for the cutoff variable.

As this graph outputs 3 attributes, create them from the Attributes tab of the visualization window.

Add an attribute with the "+" button, and rename the attribute by clicking on the name.

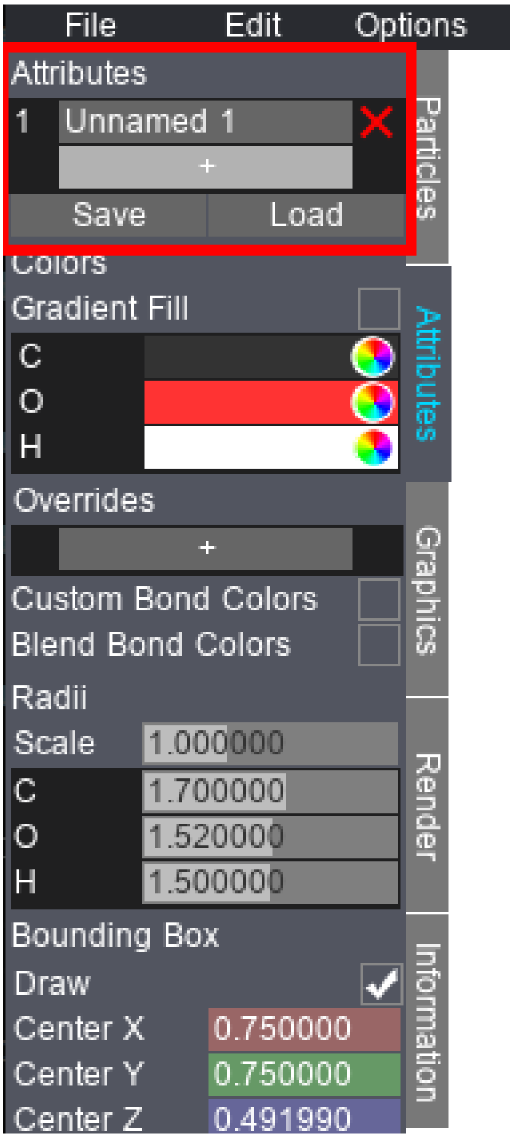

Then, associate the attribute writer node to each created attribute.

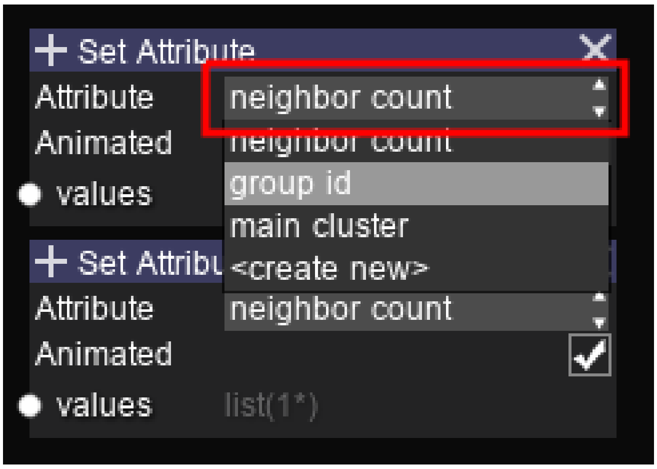

The created graph can be executed for the active snapshot by clicking on the `Run` button, or for the whole trajectory with the `Run All` button. Alternatively, and this is the simplest method to visualize the results, enable the Run on Seek option below, and the graph will be executed whenever the active frame is changed. The visualized frame can be changed with the left/right arrow keys.

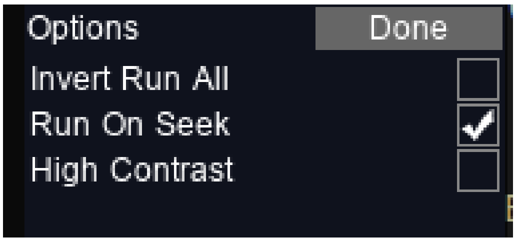

Finally, the values of an attribute can be visualized by enabling the gradient fill option, and selecting the corresponding attribute to be visualized. Note that values outside of the 0 1 range cannot be visualized properly as of version 0.12.

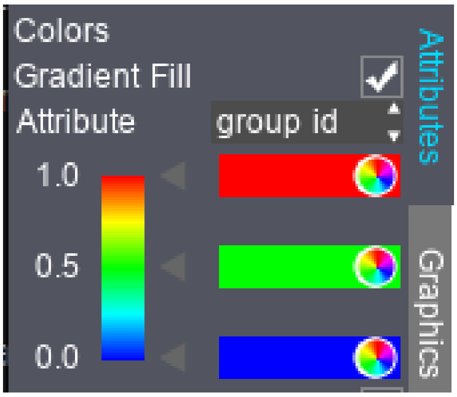

The resulting attributes, as well as the created graph can be saved and loaded for later use.
